# Patient–ventilator asynchrony, impact on clinical outcomes and effectiveness of interventions: a systematic review and meta-analysis

**DOI:** 10.1186/s40560-021-00565-5

**Published:** 2021-08-16

**Authors:** Michihito Kyo, Tatsutoshi Shimatani, Koji Hosokawa, Shunsuke Taito, Yuki Kataoka, Shinichiro Ohshimo, Nobuaki Shime

**Affiliations:** 1 Department of Emergency and Critical Care Medicine, Graduate School of Biomedical and Health Sciences, Hiroshima University, Kasumi 1-2-3, Minami-ku, Hiroshima, 734-8551 Japan; 2 Department of Anesthesiology and Reanimatology, Faculty of Medicine Sciences, University of Fukui, 23-3 Eiheijicho, Yoshidagun, Fukui, 910-1193 Japan; 3 Division of Rehabilitation, Department of Clinical Practice and Support, Hiroshima University Hospital, Kasumi 1-2-3, Minami-ku, Hiroshima, 734-8551 Japan; 4 Department of Internal Medicine, Kyoto Min-Iren Asukai Hospital, Tanaka Asukai-cho 89, Sakyo-ku, Kyoto, 606-8226 Japan; 5 Systematic Review Workshop Peer Support Group (SRWS-PSG), Osaka, Japan; 6 Section of Clinical Epidemiology, Department of Community Medicine, Kyoto University Graduate School of Medicine, Yoshida Konoe-cho, Sakyo-ku, Kyoto, 606-8501 Japan; 7 Department of Healthcare Epidemiology, Graduate School of Medicine and Public Health, Kyoto University, Yoshida Konoe-cho, Sakyo-ku, Kyoto, 606-8501 Japan

**Keywords:** Patient–ventilator interaction, Asynchrony index, Ineffective triggering, Mechanical ventilation, ICU, Mortality

## Abstract

**Background:**

Patient–ventilator asynchrony (PVA) is a common problem in patients undergoing invasive mechanical ventilation (MV) in the intensive care unit (ICU), and may accelerate lung injury and diaphragm mis-contraction. The impact of PVA on clinical outcomes has not been systematically evaluated. Effective interventions (except for closed-loop ventilation) for reducing PVA are not well established.

**Methods:**

We performed a systematic review and meta-analysis to investigate the impact of PVA on clinical outcomes in patients undergoing MV (Part A) and the effectiveness of interventions for patients undergoing MV except for closed-loop ventilation (Part B). We searched the Cochrane Central Register of Controlled Trials, MEDLINE, EMBASE, ClinicalTrials.gov, and WHO-ICTRP until August 2020. In Part A, we defined asynchrony index (AI) ≥ 10 or ineffective triggering index (ITI) ≥ 10 as high PVA. We compared patients having high PVA with those having low PVA.

**Results:**

Eight studies in Part A and eight trials in Part B fulfilled the eligibility criteria. In Part A, five studies were related to the AI and three studies were related to the ITI. High PVA may be associated with longer duration of mechanical ventilation (mean difference, 5.16 days; 95% confidence interval [CI], 2.38 to 7.94; *n* = 8; certainty of evidence [CoE], low), higher ICU mortality (odds ratio [OR], 2.73; 95% CI 1.76 to 4.24; *n* = 6; CoE, low), and higher hospital mortality (OR, 1.94; 95% CI 1.14 to 3.30; *n* = 5; CoE, low). In Part B, interventions involving MV mode, tidal volume, and pressure-support level were associated with reduced PVA. Sedation protocol, sedation depth, and sedation with dexmedetomidine rather than propofol were also associated with reduced PVA.

**Conclusions:**

PVA may be associated with longer MV duration, higher ICU mortality, and higher hospital mortality. Physicians may consider monitoring PVA and adjusting ventilator settings and sedatives to reduce PVA. Further studies with adjustment for confounding factors are warranted to determine the impact of PVA on clinical outcomes.

*Trial registration* protocols.io (URL: https://www.protocols.io/view/the-impact-of-patient-ventilator-asynchrony-in-adu-bsqtndwn, 08/27/2020).

**Supplementary Information:**

The online version contains supplementary material available at 10.1186/s40560-021-00565-5.

## Introduction

Patient–ventilator asynchrony (PVA) is defined as a mismatch between the breathing efforts of a patient and breath delivery by a ventilator [1]. It is a common problem in mechanically ventilated patients and has an incidence of up to 80% [2]. PVA may cause ventilator-induced lung injury due to excessive tidal volume [3, 4], and diaphragm injury from eccentric contractions [5], both of which can affect clinical outcomes.

The impact of PVA in patients undergoing mechanical ventilation on clinical outcomes appears inconsistent among studies. Thille et al. reported that higher incidence of PVA was associated with a longer duration of mechanical ventilation, but was not associated with increased mortality [6]. Conversely, Blanch et al. found that patients with higher incidence of PVA had significantly higher ICU mortality than patients with lower incidence of PVA, while the duration of mechanical ventilation did not differ significantly between the two groups [7]. It also remains unclear whether PVA itself worsens clinical outcomes [8].

Recently, closed-loop ventilation systems such as neurally adjusted ventilatory assist (NAVA) and proportional assist ventilation (PAV) were shown to decrease the incidence of PVA during the weaning phase of mechanical ventilation in many trials [9, 10]. However, these ventilator modes cannot be utilized for all patients undergoing mechanical ventilation, because they are only available in limited numbers of ventilator systems. Other respiratory management procedures such as adjustment of sedatives or ventilator settings are possibly effective for reducing PVA. Therefore, systematic summarizations of the interventions for PVA are needed to improve the clinical outcomes of patients undergoing mechanical ventilation.

We addressed two research questions in this systematic review and meta-analysis. In Part A, we addressed the impact of PVA on clinical outcomes in patients undergoing invasive mechanical ventilation. In Part B, we addressed the impact of interventions except closed-loop ventilation in patients undergoing invasive mechanical ventilation on PVA.

## Materials and methods

### Protocol and registration

We performed a systematic review and meta-analysis in accordance with the Preferred Reporting Items for Systematic Reviews and Meta-analyses (PRISMA) guidelines [11] (Additional file 1). Our protocol was registered in protocols.io (https://www.protocols.io/view/the-impact-of-patient-ventilator-asynchrony-in-adu-bsqtndwn).

### Study inclusion and exclusion criteria

The studies had to include adult patients undergoing invasive mechanical ventilation. In Part A, we defined an asynchrony index (AI) ≥ 10 or ineffective triggering index (ITI) ≥ 10 as high PVA. AI was defined as the number of asynchronous breaths, divided by the total number of breaths (both requested and delivered) multiplied by 100 [12]. ITI was defined as the number of ineffectively triggered breaths divided by the total number of triggered and ineffectively triggered breaths multiplied by 100 [13]. The counts of asynchronous breaths were set according to each study. We compared patients having high PVA with those having low PVA. We included published and unpublished observational studies, as well as secondary analyses of randomized controlled trials (RCTs) comprising cross-over trials, cluster-randomized trials, and quasi-randomized trials. In Part B, we assessed the effectiveness of patient management procedures for PVA on clinical outcomes including reduced PVA. We included published and unpublished interventional studies, as well as RCTs comprising cross-over trials, cluster-randomized trials, and quasi-randomized trials.

In Part A, we excluded studies involving patients who were only post-surgery, suspected of having bronchopleural fistulas or air leaks, and aged less than 18 years. In Part B, we excluded studies evaluating the effects of interventions of closed-loop ventilation systems, such as NAVA, PAV and SmartCare^®^.

### Outcomes of interest

*Part A*. The primary outcomes were duration of mechanical ventilation, ICU mortality, and hospital mortality, and the secondary outcomes were incidence of reintubation and incidence of tracheostomy.

*Part B*. The primary outcomes were incidence of PVA and duration of mechanical ventilation, and the secondary outcomes were ICU mortality, hospital mortality, incidence of reintubation, and incidence of tracheostomy.

### Search strategy

We searched MEDLINE, EMBASE, Cochrane Central Register of Controlled Trials (CENTRAL), clinicaltrials.gov, and World Health Organization International Clinical Trials Platform Search Portal (ICTRP) with no language restrictions for studies undertaken before 07 August 2020 (Additional file 2).

### Study selection and data extraction

Two authors (MK and TS) independently assessed the remaining abstracts and, if necessary, their full-text articles to determine whether they satisfied the inclusion criteria. If two authors were unsure whether a study met the inclusion criteria, we contacted the study’s original authors and requested additional information. The two authors then compared their lists. Any differences in opinion were resolved by discussion or, if this failed, through arbitration by a third author (ST).

### Quality assessment

Two authors (MK and TS) independently assessed the risk of bias for each study by using the Quality In Prognosis Studies (QUIPS) tool [14] in Part A, and the Risk Of Bias In Non-randomized Studies—of Interventions (ROBINS-I) [15] and the Risk Of Bias tool for randomized trials (RoB2) [16] in Part B. Two authors assessed each domain by the confounding factors of age, severity score, and coexisting diseases (acute respiratory distress syndrome [ARDS], sepsis, chronic obstructive pulmonary disease, and heart failure). Any conflicts between the two authors were resolved through discussion.

### Data synthesis and statistical analysis

#### Data synthesis

All analyses were performed using Review Manager (RevMan 5.4; Nordic Cochrane Centre, Cochrane Collaboration, Copenhagen, Denmark) software. We used a random-effect model weighted by the inverse variance estimate. The effects for the continuous outcomes of duration of mechanical ventilation and AI were expressed as the mean difference (MD) with 95% confidence interval (CI). The effects for the dichotomous outcomes of mortality, incidence of reintubation, and incidence of tracheostomy were expressed as the odds ratio (OR) with 95% CI. We converted medians and interquartile ranges to means and standard deviations using a method proposed by Wan et al. [17].

#### Subgroup and sensitivity analysis

We added a subgroup analysis for the assessment of PVA represented as AI and ITI to planned subgroup analyses. We planned to carry out a sensitivity analysis for hospital mortality that was not clearly defined at a time point.

#### Assessment of heterogeneity

We calculated *I*^2^ as a measure of variation across studies that arose through heterogeneity rather than by chance, and interpreted the values as follows: 0%–40%, negligible heterogeneity; 30%–60%, mild-to-moderate heterogeneity; 50%–90%, moderate-to-substantial heterogeneity; 75%–100%, considerable heterogeneity. If heterogeneity was identified for an outcome (*I*^2^ > 50%), we investigated the underlying reasons and conduct a χ^2^ test, with a *p* value of < 0.10 considered to indicate statistical significance.

#### Assessment of publication bias

We searched the trial registers (World Health Organization International Clinical Trials Platform Search Portal and ClinicalTrials.gov) to identify completed, but unpublished, trials at the time of the review.

#### Summary of findings

In Part A, we created a summary-of-findings table that included an overall grading of the certainty of the evidence for each of the main outcomes, which was evaluated using the Grading of Recommendations, Assessment, Development and Evaluation (GRADE) approach [18].

### Statements

We followed the informative statements regarding the manner in which to communicate the findings according to the GRADE guideline [19].

## Results

### Results of the search

**Fig. 1:**
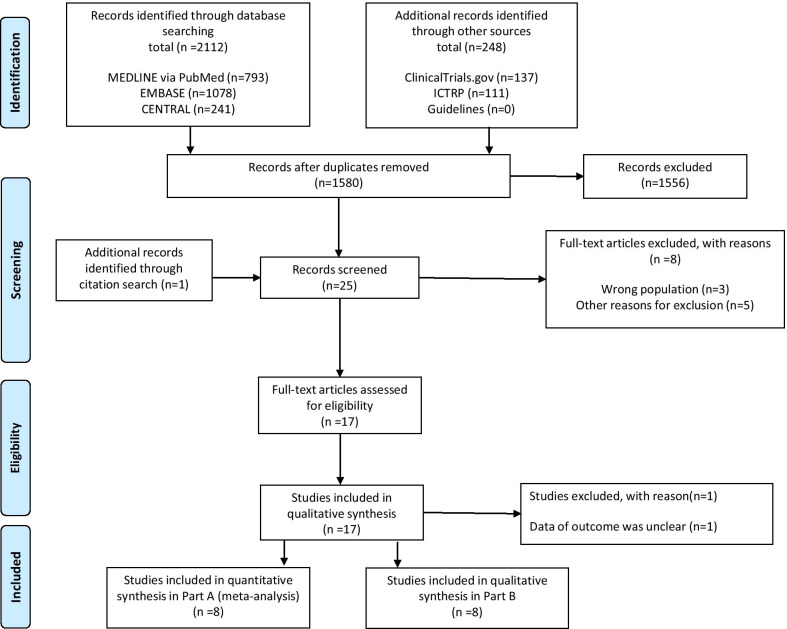
Preferred Reporting Items for Systematic Reviews and Meta-analysis (PRISMA) flowchart: results of the search strategy

We screened 1580 records, after removal of duplicates, and assessed the full-text articles of 25 studies for eligibility. Of these, eight studies [7, 12, 13, 20–24] in Part A and eight trials [6, 25–31] in Part B met the inclusion criteria (Fig. 1, Additional file 3). The search did not reveal any ongoing and unpublished studies.

### Part A (impact of PVA on clinical outcomes)

#### Characteristics of the studies included in the qualitative synthesis

Table 1 shows the characteristics of the eight included observational studies related to PVA, of which five studies were related to AI [7, 12, 21–23] and three studies were related to ITI [13, 20, 24]. According to the risk of bias in the included studies using the QUIPS tool, bias domain 6 (statistical analysis and reporting) was high in all studies except for hospital mortality in two studies (Additional file 4).

**Table 1:** Characteristics of the studies included in Part A

First author	Published year	Study design	Study location	Number of samples	Assessor	Observation duration	Index of patient–ventilator asynchrony
Thille	2006	Prospective observational	France	62	Human	30 min	Asynchrony index
de Wit	2009	Prospective observational	United States	60	Human	10 min	Ineffective trigger index
Hassan	2011	Prospective observational	Egypt	150	Human	10 min	Ineffective trigger index
Robinson	2013	Prospective observational	United States	35	Human	> 30 min	Asynchrony index
Blanch	2015	Prospective observational	Spain	50	Software	From admission until liberation from ventilator or death	Asynchrony index
Rolland-Debord	2017	Ancillary study of randomized controlled trial	France	103	Human	> 20 min	Asynchrony index
Vaporidi	2017	Prospective observational	Greece	110	Software	24 h	Ineffective trigger index
Sousa	2020	Prospective observational	Brazil	103	Software	From study inclusion until liberation from ventilator	Asynchrony index

### Results of the synthesis

**Fig. 2:**
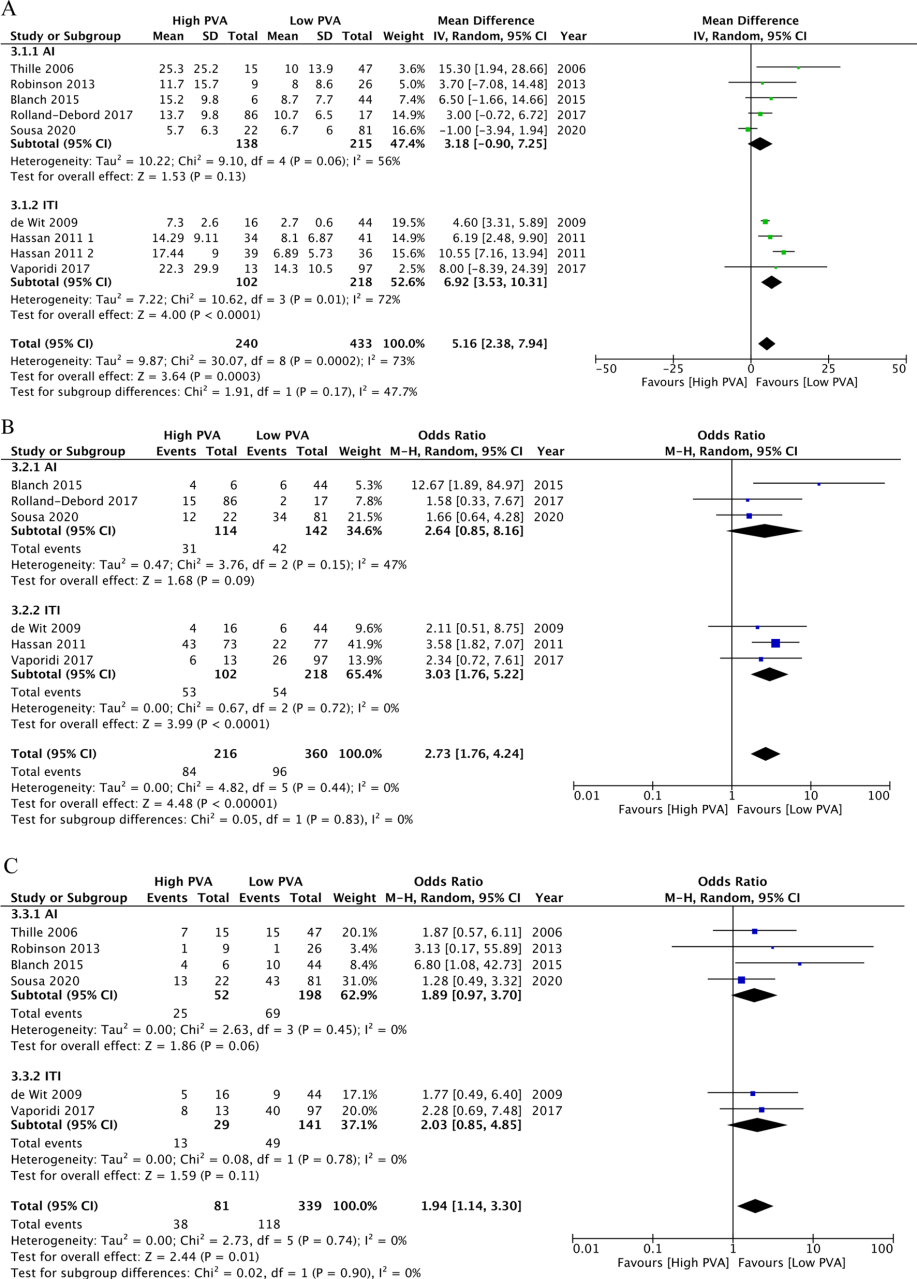

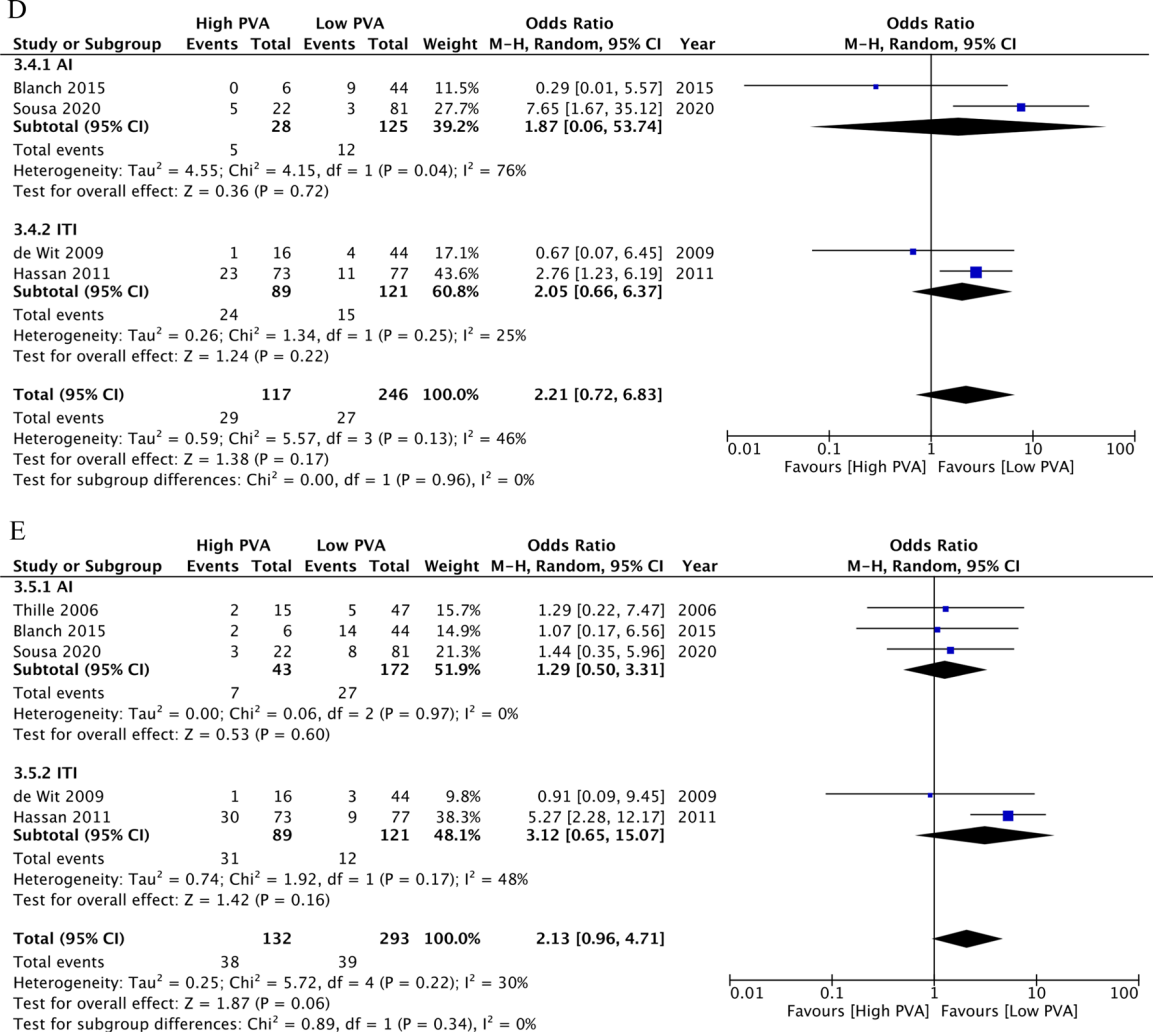
Forest plots for ventilated patients with high patient–ventilator asynchrony (PVA) versus low PVA and clinical outcomes in Part A. **A** Duration of mechanical ventilation. **B** ICU mortality. **C** Hospital mortality. **D** Incidence of reintubation. E, Incidence of tracheostomy. PVA, patient–ventilator asynchrony; AI, asynchrony index; ITI, ineffective triggering index; SD, standard deviation; CI, confidence interval; IV, inverse variance; M–H, Mantel–Haenszel

The meta-analyses for the associations of PVA with the primary and secondary outcomes are shown in Table 2 and Fig. 2. Regarding the primary outcomes, high PVA may be associated with longer duration of mechanical ventilation (MD, 5.16 days; 95% CI 2.38 to 7.94; *n* = 8; CoE, low), higher ICU mortality (OR, 2.73; 95% CI 1.76 to 4.24; *n* = 6; CoE, low), and higher hospital mortality (OR, 1.94; 95% CI 1.14 to 3.30; *n* = 5; CoE, low). Regarding the secondary outcomes, high PVA may be associated with higher incidence of reintubation (OR, 2.21; 95% CI 0.72 to 6.83; *n* = 4; CoE, low) and higher incidence of tracheostomy (OR, 2.13; 95% CI 0.96 to 4.71; *n* = 5; CoE, low).

**Table 2:** Summary of findings in the eight studies focused on patient–ventilator asynchrony in ventilated patients in Part A

Overview of study design						
Patients or study population: adult patients requiring mechanical ventilation in the ICU Exposure: high patient–ventilator asynchrony Comparison: low patient–ventilator asynchrony						
Outcome	Illustrative comparative risks^d^ (95% CI)		Relative effect (95% CI)	No. of participants (studies)	Certainty of the evidence (GRADE)	Comments
	Assumed risk	Corresponding risk				
	Control	Intervention				
Duration of mechanical ventilation (days)	Study population		–	673 (8 studies)	⊕⊕⊝⊝ Low^a,b^	
		MD: 5.16 (2.38 to 7.94)				
ICU mortality	Study population		OR2.73 (1.76 to 4.24)	576 (6 studies)	⊕⊕⊝⊝ Low^a,b^	
	267 per 1000	498 per 1000 (390 to 607)				
Hospital mortality	Study population		OR1.94 (1.14 to 3.30)	420 (6 studies)	⊕⊕⊝⊝ Low^a,b^	
	348 per 1000	509 per 1000 (378 to 638)				
Incidence of reintubation	Study population		OR2.21 (0.72 to 8.83)	363 (4 studies)	⊕⊕⊝⊝ Low^a,c^	
	110 per 1000	214 per 1000 (82 to 457)				
Incidence of tracheostomy	Study population		OR2.13 (0.96 to 4.71)	425 (5 studies)	⊕⊕⊝⊝ Low^a,c^	
	133 per 1000	246 per 1000 (128 to 420)				

GRADE Working Group grades of evidence

High certainty: we are very confident that the true effect lies close to the estimate of the effect

Moderate certainty: we are moderately confident in the effect estimate; the true effect is likely to be close to the estimate of the effect, but there is a possibility that it is substantially different

Low certainty: our confidence in the effect estimate is limited; the true effect may be substantially different from the estimate of the effect

Very low certainty: we have very little confidence in the effect estimate; the true effect is likely to be substantially different from the estimate of effect

CI, confidence interval; ICU, intensive care unit; MD, mean difference; OR, odds ratio

^a^Downgraded one point because of a high risk of bias associated with statistical analysis and reporting

^b^Downgraded one point because of imprecise (optimal information size)

^c^Downgraded one point because of imprecise (confidence interval)

^d^The corresponding risk (and its 95% CI) is based on the assumed risk in the comparison group and the relative effect (and its 95% CI) estimated for the intervention group. Assumed risk was estimated from the meta-analysis of control risks

### Subgroup and sensitivity analysis

We conducted the added and prescribed subgroup analysis for the index of PVA (AI/ITI) and the method (human/software) of PVA assessment (Fig. 2, Additional file 5). Regarding the primary outcomes, AI ≥ 10 may be associated with longer duration of mechanical ventilation (MD, 3.18 days; 95% CI − 0.90 to 7.25; *n* = 5), higher ICU mortality (OR, 2.64; 95% CI 0.85 to 8.16; *n* = 3), and higher hospital mortality (OR, 1.89; 95% CI 0.97 to 3.70; *n* = 4). ITI ≥ 10 may be associated with longer duration of mechanical ventilation (MD 6.92 days; 95% CI 3.53 to 10.31; *n* = 3), higher ICU mortality (OR, 3.03; 95% CI 1.76 to 5.22; *n* = 3), and higher hospital mortality (OR, 2.03; 95% CI 0.85 to 4.85; *n* = 2). Studies that focused on the duration of mechanical ventilation had a similar MD for the relationship between human and software assessments (human assessment: MD, 6.21 days; 95% CI 3.49 to 8.93 versus software assessment: MD, 2.30 days; 95% CI −3.76 to 8.35, *P* = 0.25). Studies that focused on ICU mortality and hospital mortality also had a similar OR for the relationship between human and software assessments (human assessment: OR, 2.96; 95% CI 1.67–5.23 compared to software assessment: OR, 2.79; 95% CI, 1.06–7.38, *P* = 0.92; human assessment: OR, 1.90; 95% CI 0.83–4.39 compared to software assessment: OR, 2.09; 95% CI 0.92–4.71, *P* = 0.88, respectively).

### Difference between protocol and review

We did not perform predetermined subgroup analyses for the following variables due to insufficient data: causes of admission to ICU (internal diseases versus traumatic diseases), coexisting ARDS (ARDS versus non-ARDS), ventilator mode (assist control mode versus pressure-support ventilation), and timing (acute phase versus whole period of mechanical ventilation). We were also unable to perform the following planned sensitivity analyses for the primary outcomes due to insufficient data: exclusion of studies (i) using imputed statistics; (ii) including timing when assessing of PVA was not only acute phase, but also outside the acute phase; (iii) including post-operative patients, and (iv) with high or moderate risk of bias, due to insufficient data.

### Part B (interventions for reducing PVA)

#### Characteristic of the studies included in the qualitative synthesis

The characteristics of the eight included trials, of which four trials were related to ventilator settings [6, 28–30], three trials were related to sedation [25, 27, 31], and one trial was related to ventilator settings and sedation [26], are shown in Table 3. The risks of bias using the ROBINS-I and RoB2 tools are shown in Additional files 6, 7 and 8.

**Table 3:** Characteristics and outcomes of the included trials in Part B

First author	Published year	Study design	Study location	Number of participants	Inclusion criteria	Intervention/comparison	Observation duration	Outcome	Results
Thille	2008	Non-randomized interventional study	France	12	Intubated patients with greater than 10% ineffective breaths while receiving PSV	1) Baseline: without PEEP and after application of 5 cm H_2_O of external PEEP 2) Gradual decrease in pressure-support level 3) Gradual reduction in insufflation time	10 min	Asynchrony index	Optimization of the pressure-support level decreased the asynchrony index from 45% (36%–52%) to 0% (0%–7%, *P* < 0.01). Reducing the insufflation time decreased the asynchrony index from 45% (36%–52%) to 7% (3%–15%, *P* < 0.01)
Doorduin	2015	Randomized cross-over trial	Netherlands	12	Patients with ARDS who received mechanical ventilation	1) PCV 2) PSV 3) NAVA	30 min	Dyssynchrony	Percentage of dyssynchrony breaths was significantly higher with PCV than with PSV.
Figueroa-Casas	2016	Non-randomized interventional study	United States	19	Patients with ARDS who received mechanical ventilation for less than 72 hours, with expectation to continue it for at least 48 hours	1) On volume assist control mode, each with set tidal volume of 6, 7.5, and 9 ml/kg predicted body weight, respectively 2) On adaptive pressure-control mode, each with the same sizes of set tidal volume	10 min	Dyssynchrony index	In volume control mode, the median (interquartile range) DIs were 100% (22%–100%) at set VT of 6 ml/kg, and 78% (7%–100%) at 7.5 ml/kg, both higher than 25% (0%–45%) at 9 ml/kg (*P* = 0.02 and 0.01, respectively) In adaptive pressure-control mode, compared with volume control mode, the DIs were lower at set VT of 6 and 7.5 ml/kg (*P* = 0.004 for both)
Luo	2015	Randomized controlled trial	China	40	Patients with ARDS who received mechanical ventilation	1) SIMV + PS 2) ACV	From 24 hours after intubation to spontaneous breathing trial	Patient–ventilator asynchrony Duration of mechanical ventilation Hospital mortality	Percentage of patient–ventilator asynchrony, duration of mechanical ventilation and hospital mortality did not differ significantly between the two groups
Bassuoni	2012	Randomized controlled trial	Egypt	230	Patients who expected to require invasive mechanical ventilation for more than 48 h on admission to the surgical intensive care	1) Daily interruption of sedation 2) No sedation	Throughout mechanical ventilation	Asynchrony index	No sedation was associated with significantly lower asynchrony index
Conti	2016	Randomized controlled trial	Italy	26	Adult ICU patients who had failed one weaning trial	1) Dexmedetomidine 2) Propofol to maintain the RASS score within the range of + 1 to –2	Over 10 min	Asynchrony index	Mean AI was lower with dexmedetomidine than with propofol from 2 h onwards, although the two groups only differed significantly only at 12 h (2.68 % vs 9.10 %, *P* < 0.05)
Vaschetto	2014	Randomized cross-over trial	Italy	14	Intubated patients undergoing partial ventilatory support for a period less than or equal to 48 hours	1) No sedative infusion (patient awake) 2) Deep sedation, achieved by setting the propofol target blood concentration to obtain a BIS value of 40 3) Light sedation, corresponding to half the propofol target blood concentration used to achieve a BIS value of 40	25 min	Ineffective trigger index	In PSV, ITI did not differ significantly between wakefulness and light sedation (5.9% and 7.6%, respectively, *P* = 0.97), but significantly increased up to 21.8% with deep sedation (*P* < 0.0001 vs both wakefulness and light sedation)
Chanques	2013	Non-randomized interventional study	United States	30 (100 sequences)	Patients receiving mechanical ventilation if they had severe breath stacking defined as asynchrony index > 10%	1) No intervention 2) Increase in sedation-analgesia 3) Change in ventilator setting	5–30 min	Breath stacking Asynchrony index	Compared with baseline, the decrease of asynchrony index was greater after changing the ventilator setting (–99% [–92%, –100%]) than after increasing the sedation-analgesia (–41% [–66%, 7%], *P* < 0.001) or deciding to tolerate the asynchrony (4% [–4%, 12%], *P* < 0.001) Pressure-support ventilation and increased inspiratory time were independently associated with the reduction in asynchrony index

ACV, assist control ventilation; AI, asynchrony index; ARDS, acute respiratory distress syndrome; BIS, bispectral index; DI, dyssynchrony index; ICU, intensive care unit; ITI, ineffective trigger index; NAVA, neurally adjusted ventilatory assist; PCV, pressure-control ventilation; PEEP, positive end-expiratory pressure; PSV, pressure-support ventilation; RASS, Richmond agitation–sedation scale; SIMV, synchronized intermittent mandatory ventilation; VT, tidal volume

### Summary of the results

Because of the variety of interventions for PVA, a meta-analysis was not performed. Among four trials that assessed the effect of adjusting ventilator settings to reduce PVA, two trials [28], 30 assessed the mode of mechanical ventilation, one trial [29] assessed the tidal volume, and one trial [6] assessed the pressure-support level and insufflation time during pressure-support ventilation (PSV). These trials showed application of the PSV mode compared with the pressure-control ventilation mode, higher tidal volume ventilation, and increased pressure-support level in PSV were significantly associated with reduced PVA in patients undergoing mechanical ventilation.

Three trials assessed the effect of sedation on reducing PVA. No sedation was associated with significantly lower AI than daily interruption of sedation [25]. In PSV, wakefulness and light sedation significantly decreased ITI compared with deep sedation to obtain a bispectral index value of 40 [31]. Regarding sedatives, mean AI was lower with dexmedetomidine than with propofol [27].

One trial compared the effects of the sedation–analgesia and changes in ventilator settings on AI [26]. The decrease in AI was greater after changing the ventilator settings than after increasing the sedation–analgesia.

Interventions for sedation and ventilator settings were consistent in their tendency to reduce PVA (Additional file 9).

## Discussion

The results of the present review demonstrated that PVA, represented by AI or ITI ≥ 10, may be associated with hard outcomes including duration of mechanical ventilation, ICU mortality, and hospital mortality based on eight studies including 673 patients. Interventions for PVA, such as adjustment of sedation and ventilator settings, have the potential to reduce PVA.

The associations between PVA and longer duration of mechanical ventilation or higher mortality suggests that intensive care physicians may need to consider paying attention to PVA during management of patients undergoing invasive mechanical ventilation. The types of asynchrony reflected by the defined AI varies slightly among literatures, but mainly included ineffective triggering, double triggering, short cycling, and prolonged cycling. Ineffective triggering may be caused by increased intrinsic positive end-expiratory pressure, reduced respiratory drive, or decreased respiratory muscle strength [6, 32]. Double-triggered breaths were associated with the higher tidal volume [33], which is potentially harmful to patients on mechanical ventilation [34]. Therefore, it is very likely that a high incidence of PVA is associated with clinical outcomes. However, because the certainty of the evidence was low, mainly through a lack of adjustment for confounding factors, researchers need to perform studies with increased sample sizes and adjustment for confounding factors. Furthermore, it currently remains unknown which type of PVA has the greatest impact on the hard outcomes in patients undergoing mechanical ventilation. Moreover, reverse triggering, which has received much attention in recent years for its possible relevance to lung injury [35], was not included in many of the studies. Further research focusing on specific types of PVA including reverse triggering is needed to clarify the mechanism and impact of PVA on pulmonary pathophysiology.

To date, there is no definitive methodology for assessment of PVA. Although visual inspection of airway pressure and flow waveform is the most common approach, use of adjunctive signals such as EAdi and esophageal catheter greatly enhance the detection of PVA [36]. Software that utilizes automatic algorithms has similar power for detection of asynchronies to visual inspection expertise and EAdi signals [37]. In our subgroup analysis, the impact of PVA determined by human or software assessment on duration of mechanical ventilation and hospital mortality did not differ significantly. In the future, a standardized monitoring system that can detect PVA in real time and is easy to use in clinical and research settings will be needed.

Interventions, such as adjustment of ventilator settings and sedatives or analgesic drugs, have the potential to reduce PVA. Ventilator support needs to be adjusted to ensure that the patient’s inspiratory effort is adequate, because excessive ventilator support induces ineffective triggering through diaphragm atrophy and under assistance may result in double triggering by strong inspiratory efforts [38]. Similarly, sedatives and analgesics substantially affect the respiratory drive and PVA [2, 31, 39]. The use of dexmedetomidine and light sedation may be useful to prevent suppression of the respiratory effort, which may lead to diaphragm atrophy. Therefore, it is important to adjust the ventilator settings and sedatives while careful assessment of the patient’s inspiratory effort. Regarding the research on interventions for PVA, since there is a limited number of studies related to clinical outcomes, and thus researchers may need to consider performing more RCTs for interventions to reduce PVA and improve clinical outcomes.

The present review has several strengths. It is the first systematic review and meta-analysis to assess the effect of PVA on hard outcomes and interventions for PVA. We performed this rigorous review according to a predefined protocol using the PRISMA statement and GRADE approach. The present review also has some limitations. First, in Part A, the certainty of the evidence for all outcomes was low. Information on the associations between PVA and clinical outcomes after adjustment for confounding factors will help to clarify the impact of PVA on clinical outcomes. Second, we defined asynchrony index (AI) ≥ 10 or ineffective triggering index (ITI) ≥ 10 as high PVA. Patients in studies evaluating ITI might have various AI. However, the subgroup analysis for AI and ITI showed similar results. Third, we could not carry out several planned subgroup analyses because of the limited data. Fourth, in Part B, because of the variety and small number of interventions for PVA, a meta-analysis was not performed.

## Conclusions

PVA may be associated with clinical outcomes. Intensive care physicians may need to pay greater attention to PVA during the management of patients receiving invasive mechanical ventilation, and the potential of adjustments to ventilator settings and sedatives to reduce PVA. Future studies with larger sample sizes, adjustment for confounding factors, and focus on specific types of PVA are warranted to determine the impact of PVA on clinical outcomes. Further RCTs are also needed to clarify the effective interventions for reducing PVA.

## Supplementary Information

**Additional file 1:** PRISMA 2009 checklist.

**Additional file 2:** Search strategies.

**Additional file 3:** Characteristics of the studies excluded from the qualitative and quantitative syntheses.

**Additional file 4:** Risk of bias for each study by using the Quality In Prognosis Studies tool in Part A.

**Additional file 5:** Forest plots showing the results of subgroup analysis regarding the method (human/software) of PVA assessment (A) and sensitivity analysis for hospital mortality that was clearly defined at a time point (B) for ventilated patients with high patient–ventilator asynchrony (PVA) versus low PVA and clinical outcomes in Part A. A. 1, Duration of mechanical ventilation. A. 2, ICU mortality. A. 3, Hospital mortality. A. 4, Incidence of reintubation. A. 5, Incidence of tracheostomy. PVA, patient–ventilator asynchrony; SD, standard deviation; CI, confidence interval; IV, inverse variance; M–H, Mantel–Haenszel.

**Additional file 6:** Risk of bias for each study by using the Risk Of Bias In Non-randomized Studies - of Interventions in Part B.

**Additional file 7:** Risk of bias for each study by using the Risk Of Bias tool for randomized trials (RoB 2). Additional considerations for cross-over trials in Part B.

**Additional file 8:** Risk of bias for each study by using the Risk Of Bias tool for randomized trials (RoB 2) in Part B.

**Additional file 9:** Forest plots showing the effect of interventions for patient–ventilator asynchrony represented by the asynchrony index in Part B.

## Data Availability

The datasets used and/or analyzed during the current study are available from the corresponding author on reasonable request.

## Abbreviations

AI: Asynchrony index
ARDS: Acute respiratory distress syndrome
CoE: Certainty of evidence
GRADE: Grading of recommendations, assessment, development and evaluation
ICU: Intensive care unit
ITI: Ineffective triggering index
MD: Mean difference
NAVA: Neurally adjusted ventilatory assist
OR: Odds ratio
PAV: Proportional assist ventilation
PRISMA: Preferred reporting items for systematic reviews and meta-analyses
PSV: Pressure-support ventilation
PVA: Patient–ventilator asynchrony
QUIPS: Quality in Prognosis Studies
RCTs: Randomized controlled trials
RoB2: Risk of Bias tool for randomized trials
ROBINS-I: Risk Of Bias In Non-randomized Studies—of Interventions
