# Supplementary material for: Patient–ventilator asynchrony, impact on clinical outcomes and effectiveness of interventions: a systematic review and meta-analysis
Source: J Intensive Care. 2021 Aug 16;9:50. doi: 10.1186/s40560-021-00565-5 (PMC8365272; doi:10.1186/s40560-021-00565-5)
Supplement: Supplementary file 2 — Additional file 2: Search strategies. [file 40560_2021_565_MOESM2_ESM.docx]

Additional file 2: Search strategies

**Appendix 1: The cochrane central register of controlled trials (CENTRAL) search strategy**

#1      MeSH descriptor: [Respiration, artificial] explode all trees

#2      MeSH descriptor: [Respiratory Distress Syndrome, Adult] explode all trees

#3      MeSH descriptor: [Respiratory Insufficiency] explode all trees

#4      MeSH descriptor: [critical care] explode all trees

#5      MeSH descriptor: [intensive care units] explode all trees

#6      MeSH descriptor: [critical illness] explode all trees

#7      MeSH descriptor: [respiratory mechanics] explode all trees

#8      #1 OR #2 OR #3 OR #4 OR #5 OR #6 OR #7

#9      "mechanical ventilation":ti,ab

#10    ventilator:ti,ab

#11    "ventilator weaning":ti,ab

#12    ARDS:ti,ab

#13    "acute respiratory distress syndrome":ti,ab

#14    "respiratory failure":ti,ab

#15    "critical illness":ti,ab

#16    "intensive care unit":ti,ab

#17    "critical care":ti,ab

#18    ICU:ti,ab

#19    #9 OR #10 OR #11 OR #12 OR #13 OR #14 OR #15 OR #16 OR #17 OR #18

#20    #8 OR #19

#21  "patient ventilator asynchrony":ti,ab

#22  asynchrony:ti,ab

#23  asynchronies:ti,ab

#24  "patient ventilator dyssynchrony":ti,ab

#25  dyssynchrony:ti,ab

#26  dyssynchronies:ti,ab

#27  "asynchrony index":ti,ab

#28  "ineffective effort":ti,ab

#29  "wasted effort":ti,ab

#30  "ineffective trigger":ti,ab

#31  "double triggering":ti,ab

#32  "auto triggering":ti,ab

#33  autotriggering:ti,ab

#34  "trigger delay":ti,ab

#35  "delayed trigger":ti,ab

#36  "premature cycling":ti,ab

#37  "prolonged cycling":ti,ab

#38  "delayed cycling":ti,ab

#39  "patient-ventilator Interaction":ti,ab

#40  "reverse triggering":ti,ab

#41  #21 OR #22 OR #23 OR #24 OR #25 OR #26 OR #27 OR #28 OR #29 OR #30 OR #31

OR #32 OR #33 OR #34 OR #35 OR #36 OR #37 OR #38 OR #39 OR #40

#42  #20 AND #41

**Appendix 2: MEDLINE (via PubMed) search strategy**

#1      Respiration, artificial[mesh]

#2      respiratory distress syndrome, adult[mesh]

#3      Respiratory Insufficiency[mesh]

#4      critical care[mesh]

#5      intensive care units[mesh]

#6      critical illness[mesh]

#7      respiratory mechanics[mesh]

#8      #1 OR #2 OR #3 OR #4 OR #5 OR #6 OR #7

#9      “mechanical ventilation”[tiab]

#10    ventilator[tiab]

#11    “ventilator weaning” [tiab]

#12    “interactive ventilatory support” [tiab]

#13    ARDS[tiab]

#14    “acute respiratory distress syndrome”[tiab]

#15    “respiratory failure” [tiab]

#16    “critical illness” [tiab]

#17    “intensive care unit” [tiab]

#18    “critical care” [tiab]

#19    ICU[tiab]

#20    #9 OR #10 OR #11 OR #12 OR #13 OR #14 OR #15 OR #16 OR #17 OR #18 OR #19

#21    #8 OR #20

#22  “patient ventilator asynchrony”[tiab]

#23  asynchrony[tiab]

#24  asynchronies[tiab]

#25  "patient ventilator dyssynchrony"[tiab]

#26  dyssynchrony[tiab]

#27  dyssynchronies[tiab]

#28  “asynchrony index”[tiab]

#29  “ineffective effort”[tiab]

#30  “wasted effort”[tiab]

#31  “ineffective trigger”[tiab]

#32  “double triggering”[tiab]

#33  “auto triggering”[tiab]

#34  autotriggering[tiab]

#35  “trigger delay”[tiab]

#36  “delayed trigger”[tiab]

#37  “premature cycling”[tiab]

#38  “prolonged cycling”[tiab]

#39  “delayed cycling”[tiab]

#40  “patient-ventilator Interaction”[tiab]

#41  “reverse triggering”[tiab]

#42  #22 OR #23 OR #24 OR #25 OR #26 OR #27 OR #28 OR #29 OR #30 OR #31 OR #32

OR #33 OR #34 OR #35 OR #36 OR #37 OR #38 OR #39 OR #40 OR #41

#43  #21 AND #42

**Appendix 3. EMBASE search strategy**

S1   EMB.EXACT.EXPLODE("artificial ventilation")

S2   EMB.EXACT.EXPLODE("adult respiratory distress syndrome")

S3   EMB.EXACT.EXPLODE("acute respiratory failure")

S4   EMB.EXACT.EXPLODE("intensive care unit")

S5   EMB.EXACT.EXPLODE("critical illness")

S6   EMB.EXACT.EXPLODE("breathing mechanics")

S7   S1 OR S2 OR S3 OR S4 OR S5 OR S6

S8   ab(“mechanical ventilation”) OR ti(“mechanical ventilation”)

S9   ab(ventilator) OR ti(ventilator)

S10  ab(“ventilator weaning”) OR ti(“ventilator weaning”)

S11  ab(ARDS) OR ti(ARDS)

S12  ab(“acute respiratory distress syndrome”) OR ti(“acute respiratory distress syndrome”)

S13  ab(“respiratory failure”) OR ti(“respiratory failure”)

S14  ab(“critical illness”) OR ti(“critical illness”)

S15  ab(“intensive care unit”) OR ti(“intensive care unit”)

S16  ab(“critical care”) OR ti(“critical care”)

S17  ab(ICU) OR ti(ICU)

S18  S8 OR S9 OR S10 OR S11 OR S12 OR S13 OR S14 OR S15 OR S16 OR S17

S19  S7 OR S18

S20  ab(“patient ventilator asynchrony”) OR ti(“patient ventilator asynchrony”)

S21  ab(dyssynchrony) OR ti(dyssynchrony)

S22  ab(asynchrony) OR ti(asynchrony)

S23  ab(asynchronies) OR ti(asynchronies)

S24  ab(“asynchrony index”) OR ti(“asynchrony index”)

S25  ab(“ineffective effort”) OR ti(“ineffective effort”)

S26  ab(“wasted effort”) OR ti(“wasted effort”)

S27  ab(“ineffective trigger”) OR ti(“ineffective trigger”)

S28  ab(“double triggering”) OR ti(“double triggering”)

S29  ab(“auto triggering”) OR ti(“auto triggering”)

S30  ab(autotriggering) OR ti(autotriggering)

S31  ab(“trigger delay”) OR ti(“trigger delay”)

S32  ab(“delayed trigger”) OR ti(“delayed trigger”)

S33  ab(“premature cycling”) OR ti(“premature cycling”)

S34  ab(“prolonged cycling”) OR ti(“prolonged cycling”)

S35  ab(“delayed cycling”) OR ti(“delayed cycling”)

S36  ab(“patient-ventilator Interaction”) OR ti(“patient-ventilator Interaction”)

S37  ab( “reverse triggering”) OR ti( “reverse triggering”)

S38  S20 OR S22 OR S23 OR S24 OR S25 OR S26 OR S27 OR S28 OR S29 OR S30 OR

S31 OR S32 OR S33 OR S34 OR S35 OR S36 OR S37

S39  S38 AND S19

**Appendix 4. WHO International Clinical Trials Registry Platform (ICTRP) search strategy**

http://apps.who.int/trialsearch/

Date searched: August 2020

Advanced Search >

Condition: (“mechanical ventilation” OR ventilator OR “ventilator weaning” OR “interactive ventilatory support” OR ARDS OR “acute respiratory distress syndrome” OR “respiratory failure” OR “critical illness” OR “intensive care unit” OR “critical care” OR ICU)

Interventions: (“patient ventilator asynchrony” OR asynchrony OR asynchronies OR dyssynchrony OR “ineffective effort” OR “wasted effort” OR “ineffective trigger” OR triggering OR “trigger delay” OR “delayed trigger” OR cycling OR “patient-ventilator Interaction”)

Recruitment status: ALL

**Appendix 5: ClinicalTrials.gov search strategy**

Advanced Search >

Condition or disease: “mechanical ventilation” OR ventilator OR “ventilator weaning” OR “interactive ventilatory support” OR ARDS OR “acute respiratory distress syndrome” OR “respiratory failure” OR “critical illness” OR “intensive care unit” OR “critical care” OR ICU

Study Type: All Studies

Study Results: All Studies

Eligibility Criteria: Age group: Adult (18-64), Older Adult (65+)

Outcome Measure: “patient ventilator asynchrony” OR asynchrony OR asynchronies OR dyssynchrony OR “ineffective effort” OR “wasted effort” OR “ineffective trigger” OR triggering OR “trigger delay” OR “delayed trigger” OR cycling OR “patient-ventilator Interaction”
