# Supplementary material for: Patient–ventilator asynchrony, impact on clinical outcomes and effectiveness of interventions: a systematic review and meta-analysis
Source: J Intensive Care. 2021 Aug 16;9:50. doi: 10.1186/s40560-021-00565-5 (PMC8365272; doi:10.1186/s40560-021-00565-5)
Supplement: Supplementary file 3 — Additional file 3: Characteristics of the studies excluded from the qualitative and quantitative syntheses. [file 40560_2021_565_MOESM3_ESM.docx]

Additional file 3: Characteristics of the studies excluded from the qualitative and quantitative syntheses

| **Study** | **Reason for exclusion** |
| --- | --- |
| de Haro C, et al. *Crit Care* 2019;23(1):245. | Wrong study design (observational study) |
| Zhang L, et al. *Comput Biol Med* 2020 May;120:103721. | Wrong exposure |
| Luo XY, et al. *Ann Intensive Care* 2020;10:144. | Insufficient data for exposure |
| Zhou Y, et al. https://doi.org/10.21203/rs.3.rs-129357/v1 | Insufficient data for exposure |
| Alves SH, et al. *Ann Am Thorac Soc* 2014;11(2):186–191. | Insufficient outcome data for meta-analysis |
| Basuni AS, et al. *Saudi J Anaesth* 2014;8(4):451–455. | Insufficient outcome data for meta-analysis |
| Beloncle F, et al. *Crit Care* 2017;21(1):21. | Insufficient outcome data for meta-analysis |
| Ranieri VM, et al. *Anesthesiology* 1997;86(1):79–91. | Insufficient outcome data for meta-analysis |
