# Supplementary material for: Patient–ventilator asynchrony, impact on clinical outcomes and effectiveness of interventions: a systematic review and meta-analysis
Source: J Intensive Care. 2021 Aug 16;9:50. doi: 10.1186/s40560-021-00565-5 (PMC8365272; doi:10.1186/s40560-021-00565-5)
Supplement: Supplementary file 4 — Additional file 4: Risk of bias for each study by using the Quality In Prognosis Studies tool in Part A. [file 40560_2021_565_MOESM4_ESM.docx]

Additional file 4: Risk of bias for each study by using the Quality In Prognosis Studies tool in Part A

| **Outcome** | **Author (published year)** | **Bias domain** | | | | | |
| --- | --- | --- | --- | --- | --- | --- | --- |
|  |  | **1. Study participation** | **2. Study attrition** | **3. Prognostic factor measurement** | **4. Outcome measurement** | **5. Study confounding** | **6. Statistical analysis and reporting** |
| **Duration of mechanical ventilation** | Thille (2006) | Low | Low | Low | Low | High | High |
|  | de Wit (2009) | Low | Low | Low | Low | Moderate | High |
|  | Robinson (2013) | Moderate | Low | Low | Low | High | High |
|  | Blanch (2015) | Low | Low | Low | Low | Moderate | High |
|  | Sousa (2020) | Low | Low | Low | Low | Moderate | High |
|  | Hassan (2011) | Moderate | Moderate | High | Low | High | High |
|  | Rolland-Debord (2017) | Low | Low | Low | Low | Moderate | High |
|  | Vaporidi (2017) | Low | Low | Low | Low | Moderate | High |
| **ICU mortality** | Thille (2006) | Low | Low | Low | Low | High | High |
|  | de Wit (2009) | Low | Low | Low | Low | Moderate | High |
|  | Robinson (2013) | Moderate | Low | Low | Low | High | High |
|  | Blanch (2015) | Low | Low | Low | Low | Moderate | High |
|  | Sousa (2020) | Low | Low | Low | Low | Moderate | High |
|  | Hassan (2011) | Moderate | Moderate | High | Low | High | High |
|  | Rolland-Debord (2017) | Low | Low | Low | Low | Moderate | High |
|  | Vaporidi (2017) | Low | Low | Low | Low | Moderate | High |
| **Hospital mortality** | Thille (2006) | Low | Low | Low | High | High | High |
|  | de Wit (2009) | Low | Low | Low | Low | Moderate | High |
|  | Robinson (2013) | Moderate | Low | Low | High | High | High |
|  | Blanch (2015) | Low | Low | Low | Low | Moderate | High |
|  | Sousa (2020) | Low | Low | Low | Low | Low | Low |
|  | Vaporidi (2017) | Low | Low | Low | Low | Low | Low |
| **Rate of reintubation** | de Wit (2009) | Low | Low | Low | Low | Moderate | High |
|  | Blanch (2015) | Low | Low | Low | Low | Moderate | High |
|  | Sousa (2020) | Low | Low | Low | Low | Moderate | High |
|  | Hassan (2011) | Moderate | Moderate | High | Low | High | High |
| **Rate of tracheostomy** | Thille (2006) | Low | Low | Low | Low | High | High |
|  | de Wit (2009) | Low | Low | Low | Low | Moderate | High |
|  | Blanch (2015) | Low | Low | Low | Low | Moderate | High |
|  | Sousa (2020) | Low | Low | Low | Low | Moderate | High |
|  | Hassan (2011) | Moderate | Moderate | High | Low | High | High |
