# Supplementary figures and images for: Patient–ventilator asynchrony, impact on clinical outcomes and effectiveness of interventions: a systematic review and meta-analysis
Source: J Intensive Care. 2021 Aug 16;9:50. doi: 10.1186/s40560-021-00565-5 (PMC8365272; doi:10.1186/s40560-021-00565-5)

## Slide 1
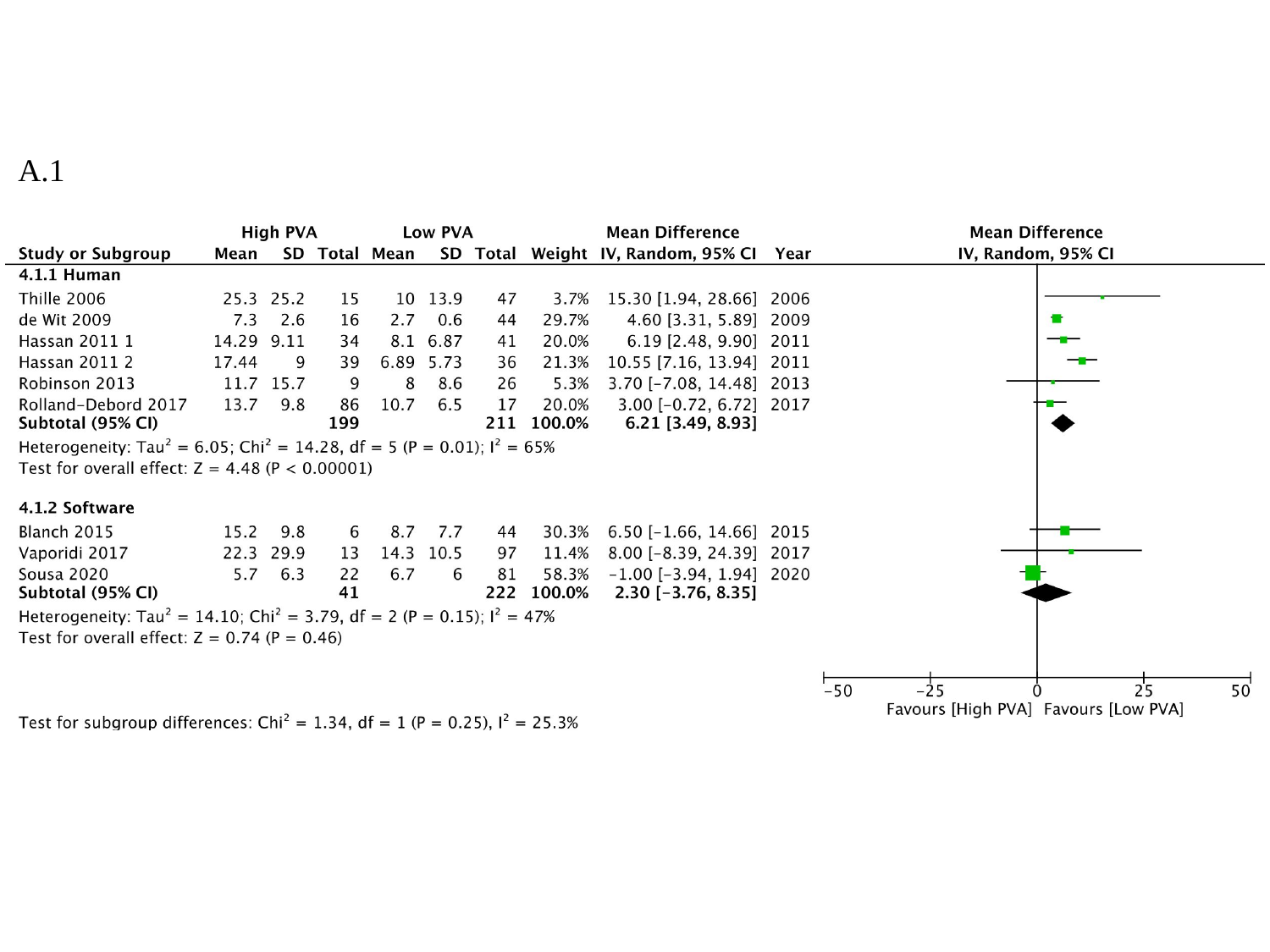

A.1

## Slide 2
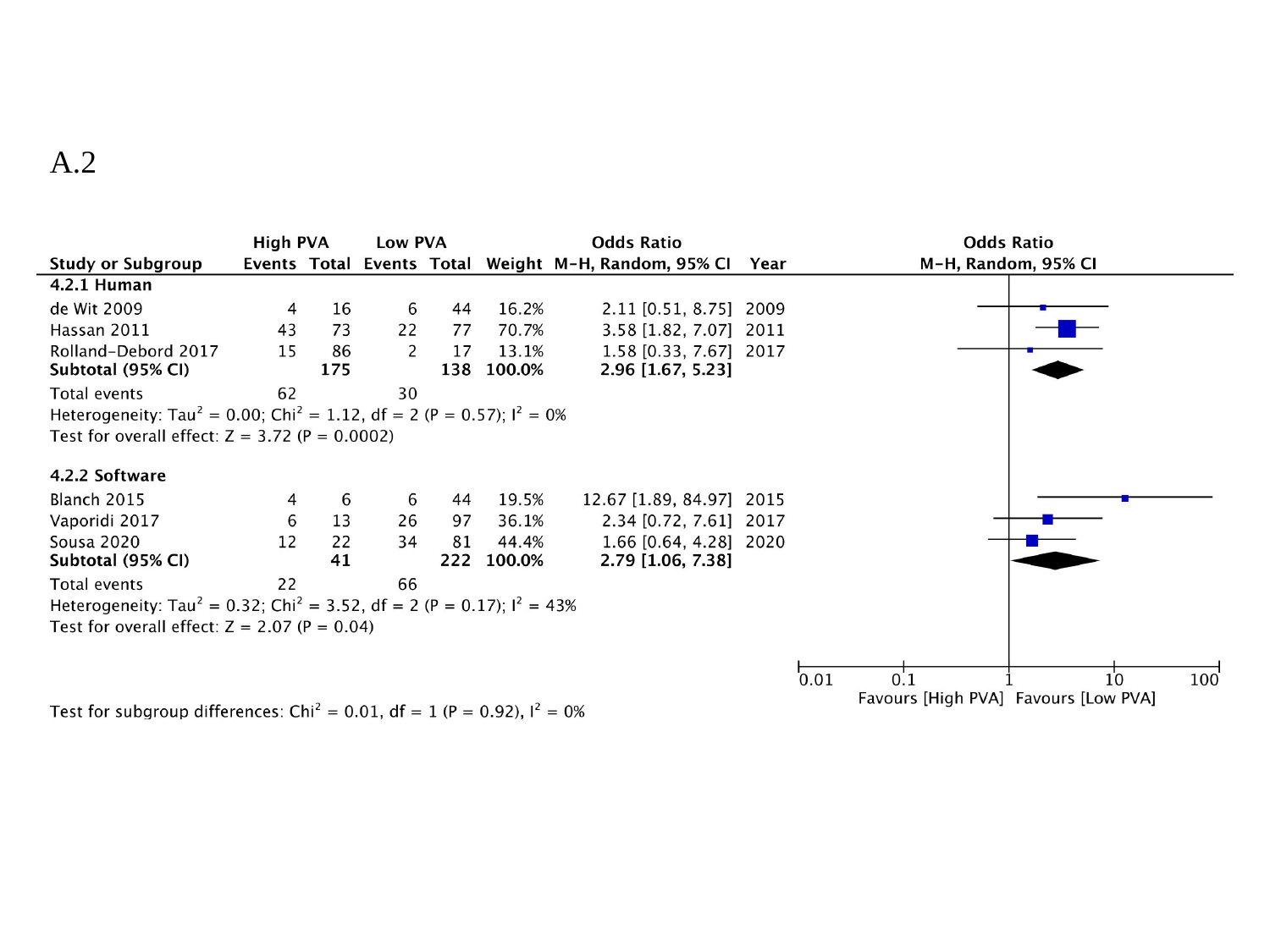

A.2

## Slide 3
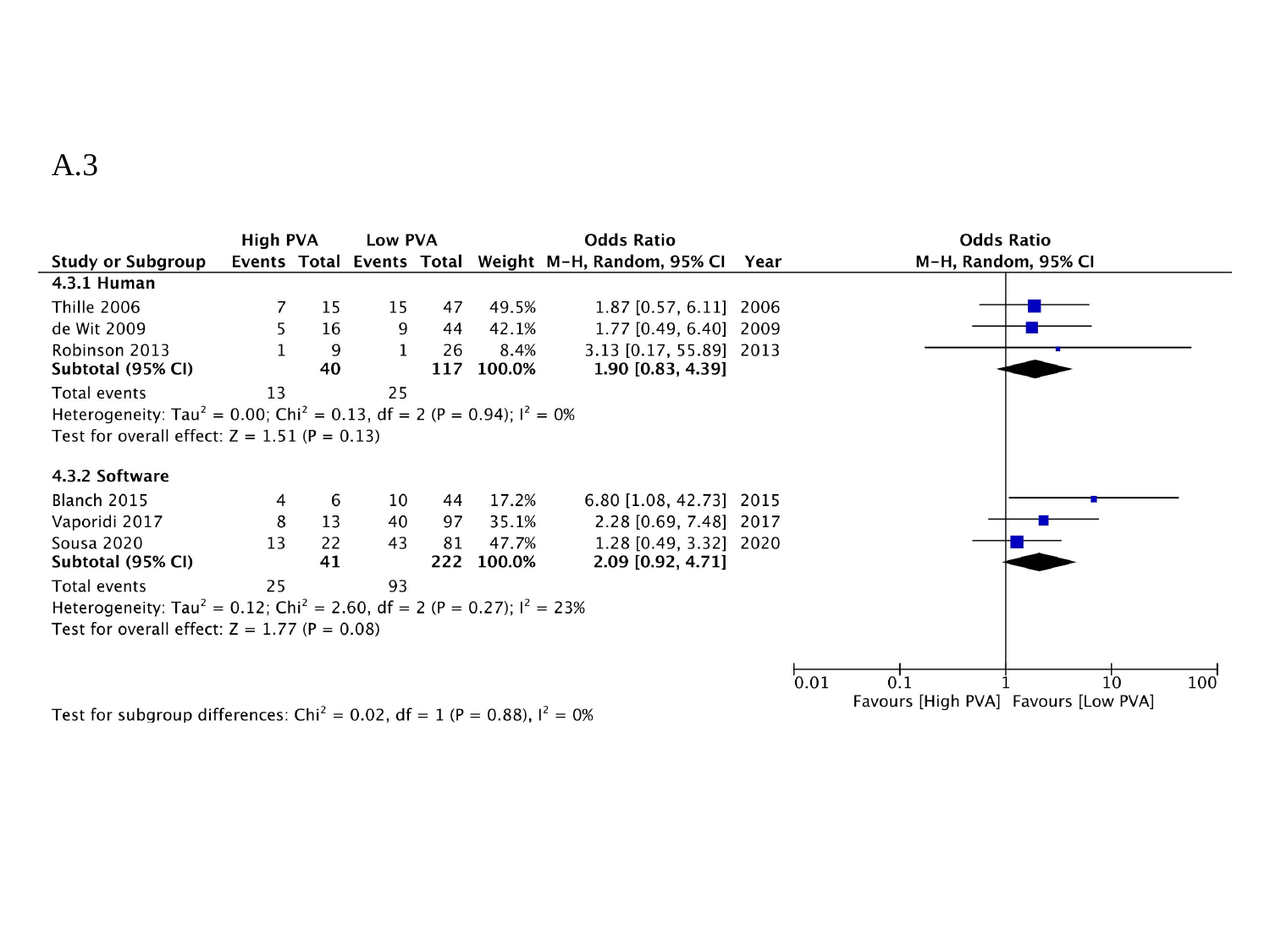

A.3

## Slide 4
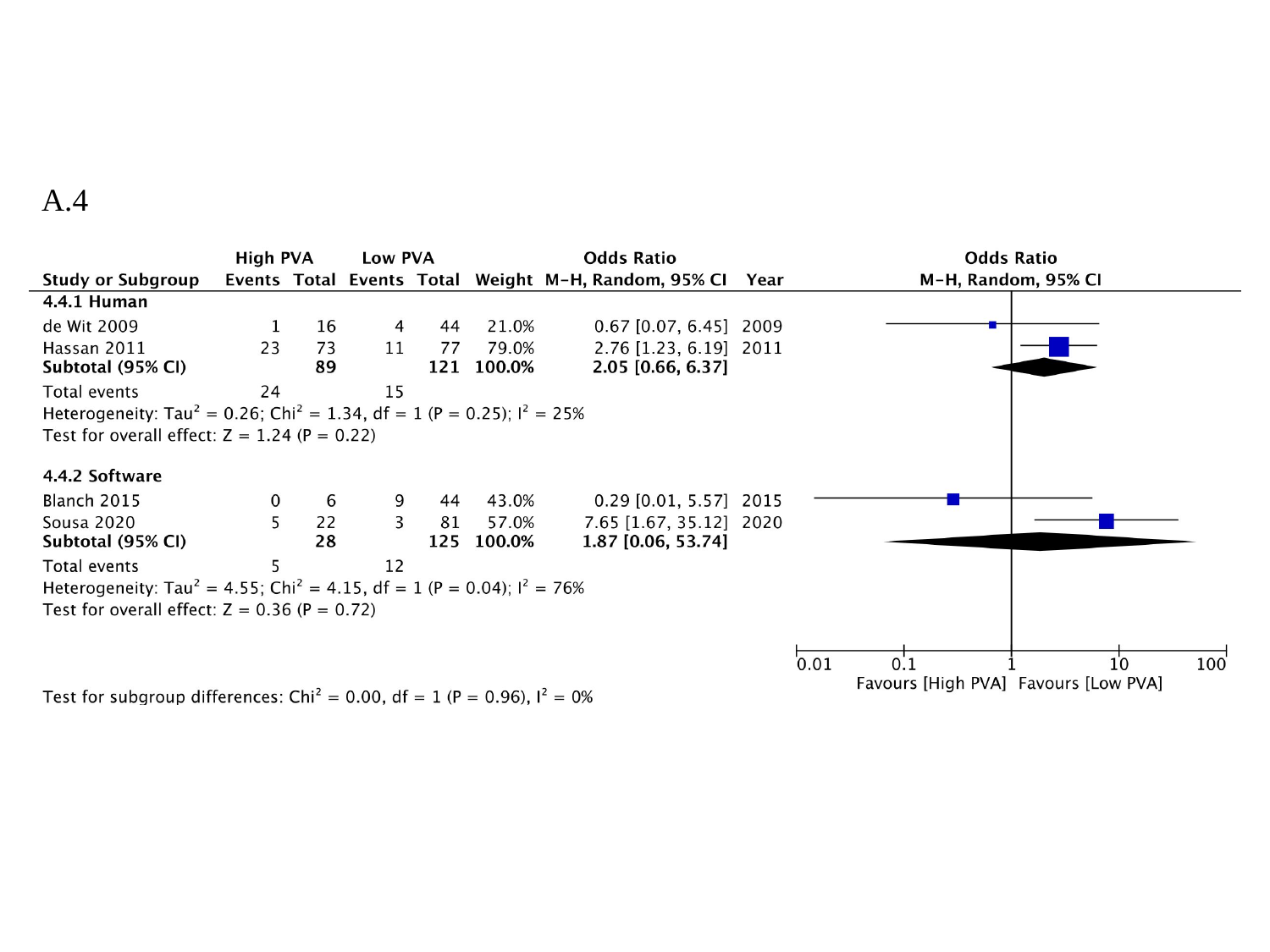

A.4

## Slide 5
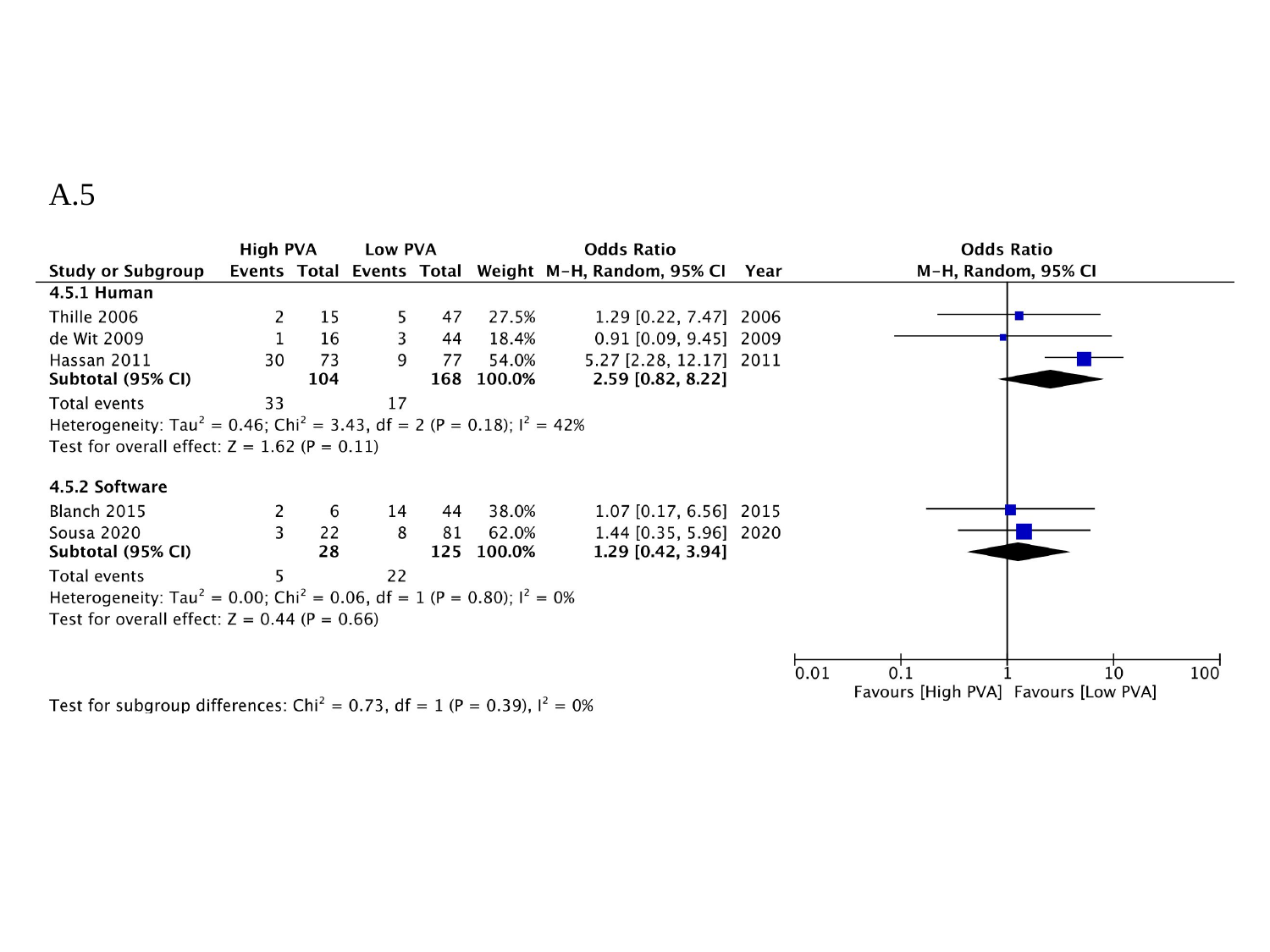

A.5

## Slide 6
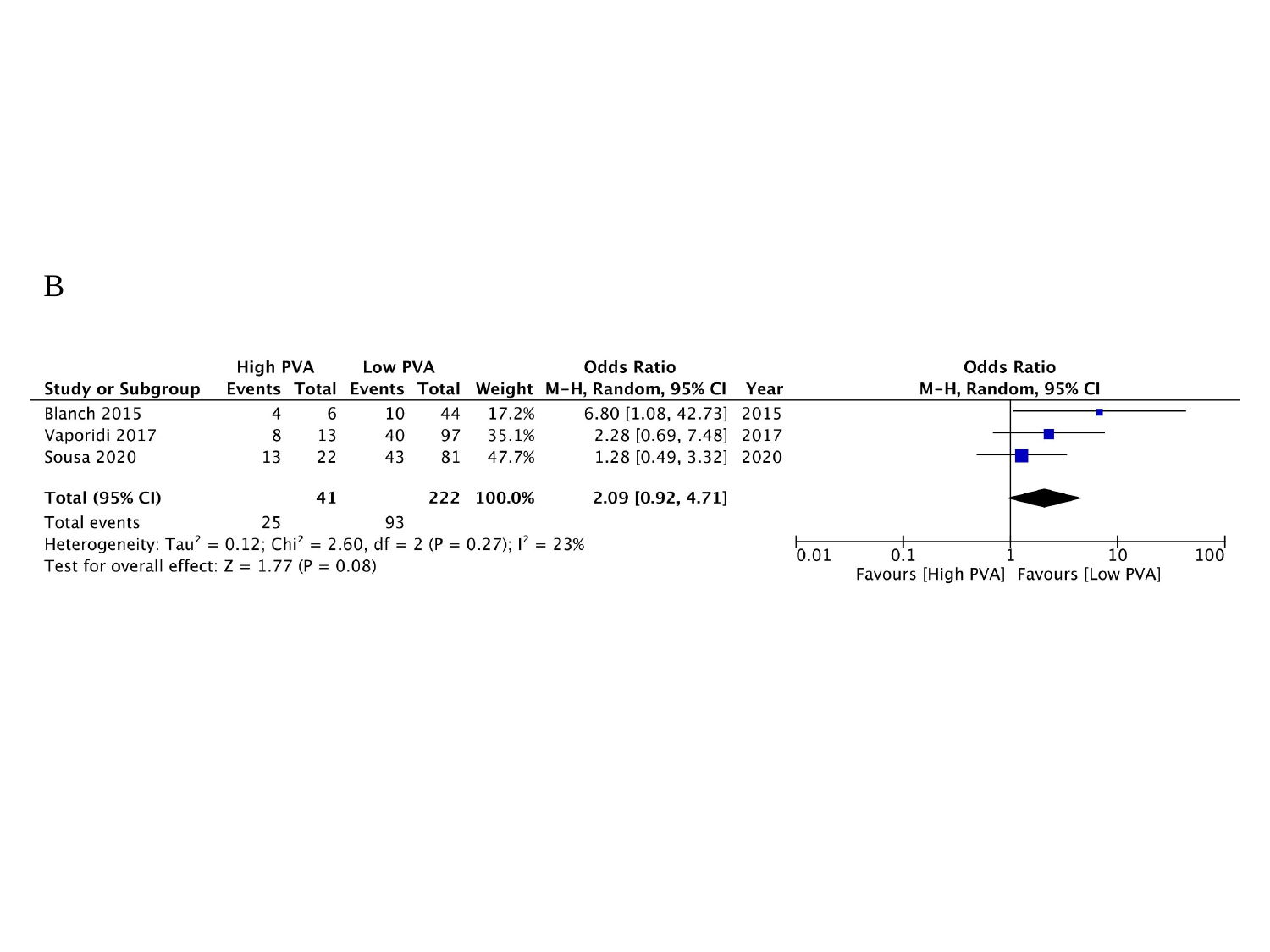

B

Supplement: Supplementary file 5 — Additional file 5: Forest plots showing the results of subgroup analysis regarding the method (human/software) of PVA assessment (A) and sensitivity analysis for hospital mortality that was clearly defined at a time point (B) for ventilated patients with high patient–ventilator asynchrony (PVA) versus low PVA and clinical outcomes in Part A. A. 1, Duration of mechanical ventilation. A. 2, ICU mortality. A. 3, Hospital mortality. A. 4, Incidence of reintubation. A. 5, Incidence of tracheostomy. PVA, patient–ventilator asynchrony; SD, standard deviation; CI, confidence interval; IV, inverse variance; M–H, Mantel–Haenszel. [file 40560_2021_565_MOESM5_ESM.pptx]

## Slide 1
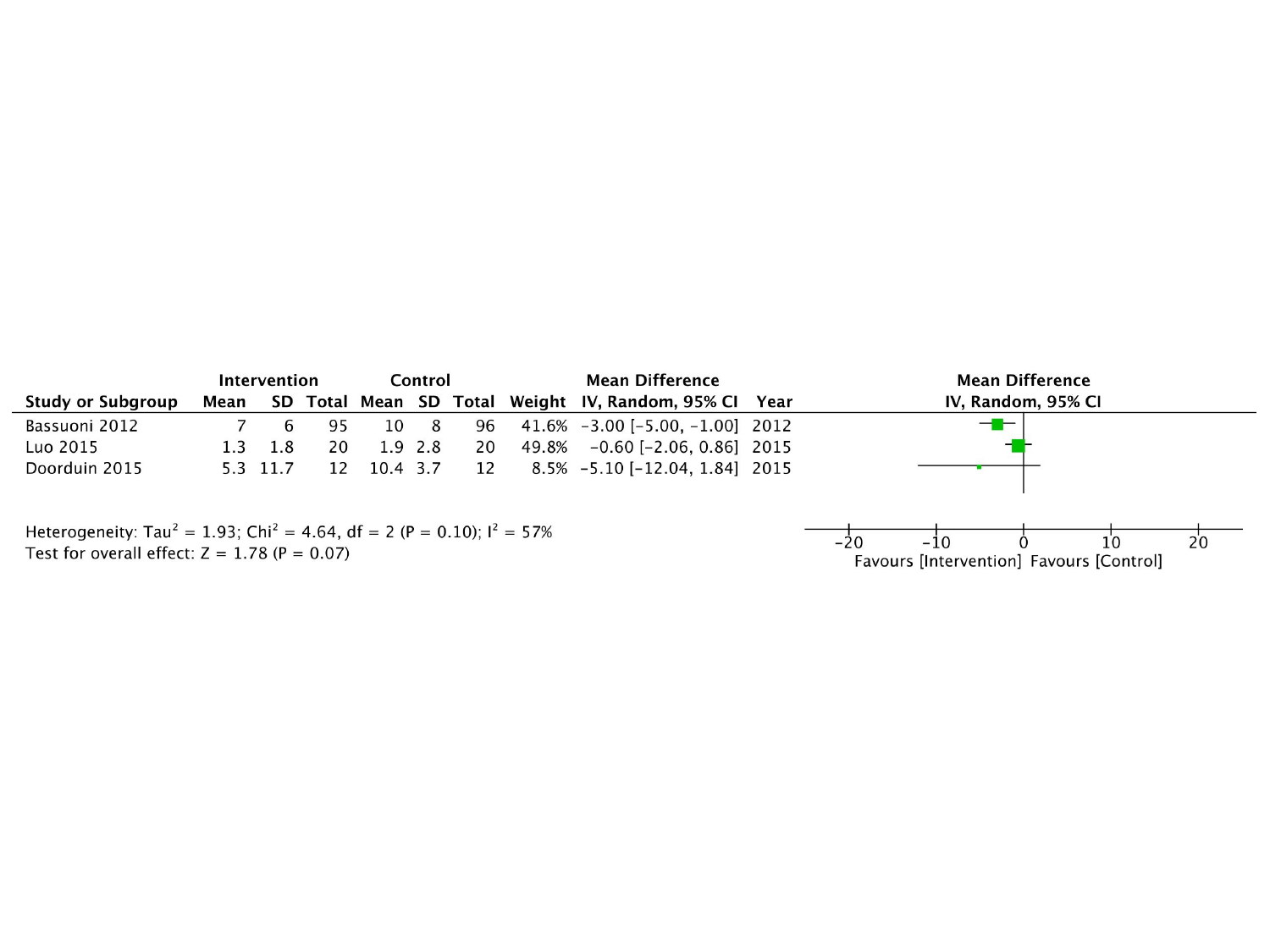

Supplement: Supplementary file 9 — Additional file 9: Forest plots showing the effect of interventions for patient–ventilator asynchrony represented by the asynchrony index in Part B. [file 40560_2021_565_MOESM9_ESM.pptx]
