# Supplementary material for: Patient–ventilator asynchrony, impact on clinical outcomes and effectiveness of interventions: a systematic review and meta-analysis
Source: J Intensive Care. 2021 Aug 16;9:50. doi: 10.1186/s40560-021-00565-5 (PMC8365272; doi:10.1186/s40560-021-00565-5)
Supplement: Supplementary file 6 — Additional file 6: Risk of bias for each study by using the Risk Of Bias In Non-randomized Studies - of Interventions in Part B. [file 40560_2021_565_MOESM6_ESM.docx]

Additional file 6: Risk of bias for each study by using the Risk Of Bias In Non-randomized Studies - of Interventions in Part B

| **Outcome** | **Author (published year)** | **Bias domain** | | | | | | |  |
| --- | --- | --- | --- | --- | --- | --- | --- | --- | --- |
|  |  | **1. Confounding** | **2. Selection of participants into the study** | **3. Classification of interventions** | **4. Deviations from intended interventions** | **5. Missing data** | **6. Measurement of outcomes** | **7. Reported results** | **Overall bias** |
| **Asynchrony** | Chanques (2013) | Low | Low | Moderate | Low | Low | Moderate | Low | Low |
|  | Figueroa-Casas (2016) | Critical | Low | Low | Low | Low | Serious | Low | Moderate |
|  | Thille (2008) | Low | Low | Low | Low | Low | Moderate | Low | Low |
