# Supplementary material for: Patient–ventilator asynchrony, impact on clinical outcomes and effectiveness of interventions: a systematic review and meta-analysis
Source: J Intensive Care. 2021 Aug 16;9:50. doi: 10.1186/s40560-021-00565-5 (PMC8365272; doi:10.1186/s40560-021-00565-5)
Supplement: Supplementary file 7 — Additional file 7: Risk of bias for each study by using the Risk Of Bias tool for randomized trials (RoB 2). Additional considerations for cross-over trials in Part B. [file 40560_2021_565_MOESM7_ESM.docx]

Additional file 7: Risk of bias for each study by using the Risk Of Bias tool for randomized trials (RoB 2). Additional considerations for crossover trials in Part B

| **Outcome** | **Author (published year)** | **Bias domain** | | | | | | |  |
| --- | --- | --- | --- | --- | --- | --- | --- | --- | --- |
|  |  | **Domain 1** | **Domain S** | **Domain 2** | | **Domain 3** | **Domain 4** | **Domain 5** | **Overall bias** |
|  |  |  |  | **For effect of assignment to intervention** | **For effect of adhering to intervention** |  |  |  |  |
| **Asynchrony** | Vaschetto (2014) | Low | Low | Low | High | Low | High | Some concerns | High |
|  | Doorduin (2015) | Some concerns | Some concerns | Low | Low | Low | Low | Some concerns | Some concerns |
