# Supplementary material for: Patient–ventilator asynchrony, impact on clinical outcomes and effectiveness of interventions: a systematic review and meta-analysis
Source: J Intensive Care. 2021 Aug 16;9:50. doi: 10.1186/s40560-021-00565-5 (PMC8365272; doi:10.1186/s40560-021-00565-5)
Supplement: Supplementary file 8 — Additional file 8: Risk of bias for each study by using the Risk Of Bias tool for randomized trials (RoB 2) in Part B. [file 40560_2021_565_MOESM8_ESM.docx]

Additional file 8: Risk of bias for each study by using the Risk Of Bias tool for randomized trials (RoB 2) in Part B

| **Outcome** | **Author (published year)** | **Bias domain** | | | | |  |
| --- | --- | --- | --- | --- | --- | --- | --- |
|  |  | **Domain 1** | **Domain 2** | **Domain 3** | **Domain 4** | **Domain 5** | **Overall bias** |
| **Asynchrony** | Bassuoni (2012) | Low | High | High | Low | Some concerns | High |
|  | Conti (2016) | Some concerns | Low | Low | Low | Low | Some concerns |
|  | Luo (2015) | Low | High | Low | High | Low | High |
| **Duration of mechanical ventilation** | Luo (2015) | Low | High | Low | Low | Low | High |
| **Hospital mortality** | Luo (2015) | Low | High | Low | Low | Low | High |
